# Facial Bone Reconstruction Using both Marine or Non-Marine Bone Substitutes: Evaluation of Current Outcomes in a Systematic Literature Review

**DOI:** 10.3390/md16010027

**Published:** 2018-01-13

**Authors:** Marco Cicciù, Gabriele Cervino, Alan Scott Herford, Fausto Famà, Ennio Bramanti, Luca Fiorillo, Floriana Lauritano, Sergio Sambataro, Giuseppe Troiano, Luigi Laino

**Affiliations:** 1 Department of Biomedical and Dental Sciences and Morphological and Functional Imaging, Messina of University, 98100 Messina, Italy; gcervino@unime.it (G.C.); enniobramanti@gmail.com (E.B.); lucafiorillo@live.it (L.F.); flauritano@unime.it (F.L.); 2 Department of Maxillofacial Surgery, Loma Linda University, Loma Linda, CA 92354, USA; aherford@llu.edu; 3 Department of Human Pathology, University of Messina, 98100 Messina, Italy; famafausto@yahoo.it; 4 Private Practive COS Center, 95100 Catania, Italy; ssambataro@centrodiortodonzia.it; 5 Department of Clinical and Experimental Medicine, University of Foggia, 71121 Foggia, Italy; giuseppe.troiano@unifg.it; 6 Multidisciplinary Department of Medical-Surgical and Odontostomatological Specialties, University of Campania “Luigi Vanvitelli”, 80121 Naples, Italy; luigi.laino@unicampania.it

**Keywords:** bone grafting, bone biocompatible materials, bone regeneration, marine collagen

## Abstract

The aim of the present investigation was to systematically analyse the literature on the facial bone reconstruction defect using marine collagen or not and to evaluate a predictable treatment for their clinical management. The revision has been performed by searched MEDLINE and EMBASE databases from 2007 to 2017. Clinical trials and animal in vitro studies that had reported the application of bone substitutes or not for bone reconstruction defect and using marine collagen or other bone substitute material were recorded following Preferred Reporting Items for Systematic Reviews and Meta-Analyses (PRISMA) guidelines. The first selection involved 1201 citations. After screening and evaluation of suitability, 39 articles were added at the revision process. Numerous discrepancies among the papers about bone defects morphology, surgical protocols, and selection of biomaterials were found. All selected manuscripts considered the final clinical success after the facial bone reconstruction applying bone substitutes. However, the scientific evidence regarding the vantage of the appliance of a biomaterial versus autologous bone still remains debated. Marine collagen seems to favor the dimensional stability of the graft and it could be an excellent carrier for growth factors.

## 1. Introduction

The management of large facial bone defects is a current challenge for clinicians and surgeons. Treatment success is frequently related to the size of the defect, the quality of the soft tissue covering the defect, the decision of reconstructive method and the choice of the grafted material [1,2,3].

Numerous bone grafts’ regenerative procedures are currently available for having complete regenerative processes after bone trauma, or for favoring healing between two bones across a diseased joint, and also for obtaining new clinical function or aesthetic on site affected by disease, infection, or resection [2,3,4,5].

Facial bone augmentation procedure using autologous bone is a reliable technique, as confirmed by several studies; however, this treatment choice has integration advantages associated with several disadvantages. Autogenous bone harvested from the patient’s extra oral or intra oral sites even considered the gold standard, at the same time is related with surgical intra and post operative complications, biological cost, and patient morbidity, pain and discomfort at the bone grafting area [1,2,3,4,5,6,7].

Recently, great interest has been directed towards the application of synthetic three-dimensional biomaterials scaffolds as bone substitutes used for facial large bone defect regeneration in order to have a substantial quantity of material and to avoid a second surgery site. Those synthetic bone substitutes materials should be non-toxic, compatible with the biological systems, and bio absorbable. The biomaterial has to be a macroscopic structure that is easy for surgeons to handle. Its microstructure should be able to promote cell adhesion, proliferation and new bone formation [4,5,6,7,8]. The fundamental key parameters for an excellent biomaterial are related to its capability on replacing the natural bone extracellular matrix. Secondly, it should be able to recall the osteo-genic cells in order to lay down the bone tissue matrix, and then the biomaterial should guarantee a sufficient vascularization to meet the growing tissue nutrient supply and clearance needs. Therefore, after being placed in situ, the microscopic features of the biomaterial should influence the host by releasing osteogenic and/or genic growth factors [5,6,7,8,9,10].

Nowadays, the tissue engineering approaches to the facial bone regeneration are connected with biomaterial matrices/scaffold that favorably interact with cells. The potential benefits of using recent tissue engineering findings is fundamental today in order to limit donor site morbidity, reducing operative time, and replacing the anatomical microstructure in an attempt to restore physiological craniofacial functions [8,9,10,11]. Currently, advances in computer-aided modeling and biomaterials manufacturing help the craniofacial surgery field, which is frequently confronted with the rebuilding of three-dimensional anatomic structures on function and aesthetics. Three-dimensional models of bone defects can be realized from patient computed tomography scans, creating a customized scaffold that interacts with the defect site and re-builds the complex anatomical features [4,5,8,12,13].

Recently, the use of bone substitutes obtained from marine origins is being considered high attractive by the industry as an alternative source. In this specific field, the marine collagen can be obtained from numerous sources. Type I collagen is obtained predominantly from skin, tendon, bone and muscle (epimysium), which is the most abundant type of collagen. Marine fish collagens find applications in numerous biomedical fields. Besides its mechanical elastic properties, marine collagen exhibited good absorption characteristics with interconnectivity between pores, which allowed human Mesenchymal Stem Cells (hMSCs) to adhere and proliferate, being a good base for osteogenic differentiation [3,7,12].

The aim of this review is to screen recent papers about biomaterials applied for facial bone reconstruction in order to give the clinicians’ valuable suggestions about the possibility of replacing autologous bone graft for large reconstruction defects.

This systematic review also aimed to evaluate the potential of reconstructive marine biomaterials uses like scaffolds for growth factor in order to provide better results in comparison to others.

## 2. Materials and Methods

### 2.1. Application Protocol and Website Recording Data

A protocol including the investigation methods and the inclusion criteria for the current revision was submitted in advance and documented on the Center for Review and Dissemination (CRD) York website PROSPERO, an international prospective register of systematic reviews. The parameters and the analytic structure of the present work can be visualized relating the CRD id and code: Application number: CRD 74603.

The data of this systematic investigation observed the Preferred Reporting Items for Systematic Review accordingly with the (PRISMA) statement [14].

### 2.2. Target Questions

The next spotlight questions processed the following guidelines, parameters, and possible aims of the Patient Intervetion Control Outcome (PICO) study design:

- What are the overall treatment outcomes of reconstructive procedures using bone substitutes in the place of autologous bone?
- As an alternative focused question, does marine collagene use provide beneficial clinical outcomes applied as scaffolds for growth factors?

### 2.3. Search Strategy

The investigation method followed the examinations of electronic databases and searches by hand. A search of five electronic databases, including Ovid MEDLINE, PubMed, EMBASE, and Dentistry and Oral Sciences Source, and Biomaterials, for relevant studies published in the English language from February 2010 to September 2017 was carried out. 

Digital and searches by hand were then performed in facial bone reconstruction, biomaterials and bone graft scaffold journals from February 2010 to September 2017, including the following: (1) Journal of Craniofacial Surgery; (2) Journal Biomed. Mater. Research A; (3) International Journal of Oral and Maxillofacial Implants; (4) Clinical Oral Implants Research; (5) Implant Dentistry; (6) International Journal of Oral and Maxillofacial Surgery; (7) Journal of Oral and Maxillofacial Surgery; (8) Journal of Dental Research; (9) Biomed. Research International; (10) International Journal of Prosthodontics; (11) Acta Biomaterial.; (12) Journal of Clinical Periodontology; (13) Clinical Implant Dentistry and Related Research; (14) European Journal of Oral Implantology; (15) Facial Plastic Surgery; (16) Materials; (17) Marine Drugs; (18) Biomaterials; (19) Br. J. Oral Maxillofacial Surgery; (20) Aesthetic Plastic Surgery; (21) Ophthal. Plast. Reconstr. Surg.; (22) J. Craniomaxillofacial Surg.; (23) Arch Facial Plast Surg.; (24) Head and Face Medicine; (25) Talanta; (26) Eur. Cell Materials; (27) Mater. Sci. Eng. C Mater. Biol. Appl.; (28) Int. J. Biol. Macromol.; (29) Adv. Food Nutr. Res.; and (30) Macromol. Bioscience. The search was limited to English language articles. In-depth research of the reference lists in the recorded manuscripts was performed in order to add significant studies and to increase the sensitivity of the revision.

### 2.4. Collection Data

The Medical Subject Headings (MeSH) was applied for finding the keywords used in the present revision. The selected key words: “facial” or “face” and “bone” or “bone graft” and “reconstruction” or “biomaterial” or “biomaterials” and “marine” and “collagen” were recorded for collecting the data. 

### 2.5. Manuscript Selections

Two independent reviewers of two different universities (Messina and Naples) singularly analyzed the obtained papers in order to select inclusion and exclusion criteria as follows. Reviewers correlated their evaluations and analyzed differences through comparing the manuscripts and consulting a third experienced senor independent reviewer (Loma Linda University) when consensus could not be reached. For the stage of the full-text articles revision, a complete independent dual analysis was performed.

### 2.6. Manuscripts Selection

The manuscripts selected in the analysis involved experimental and clinical research on humans and animals published in the English language. Letters, editorials, case reports, and PhD theses were excluded.

### 2.7. Research Classifications

The method of classification included all human prospective and retrospective clinical studies, split mouth cohort studies, case-control papers, and case series manuscripts, animal investigations and literature review published between February 2010 and September 2017, on biomaterial used for facial bone reconstruction.

### 2.8. Statement of the Problem

The sentence case of “facial bone defect reconstructive graft” was searched over each selected papers; moreover, authors investigated if there was a documented bone reconstructive surgery and a biomaterial graft placed in situ [1,2,3,4,5,6,7,8,9,10].

### 2.9. Exclusion and Inclusion Criteria

The full texts of all studies related to the main revision topics were obtained for comparing the inclusion parameters:

- Investigated surgical bone regenerative procedures in patients with vertical and horizontal defect of the jaws.
- Studies involving animals in which the created bone defects were vertical and horizontal.
- Clinical human prospective or retrospective follow-up research and trials, cohort studies, case-control investigations, and case series papers with at least six months follow-up
- Animal or in vitro studies

The following exclusion parameters for manuscripts were decided as follows:

- Research treating patients with general specific diseases, heart disease, bloody pressure disease, virus infected patients, osteoporosis, immunologic disorders, uncontrolled diabetes mellitus, or other surgical risk related systemic conditions;
- Not enough information regarding the selected topic;
- Articles published prior to 1 February 2010;
- No access to the title and abstract number in the English language.

### 2.10. Strategy for Collecting Data

After the first literature analysis, the entire manuscript titles list was highlighted to exclude irrelevant publications, case reports and the non-English language publications. Then, research was not selected based on data obtained from screening just the abstracts. The final selection was performed reading the full texts of the papers in order to approve each study’s eligibility, based on the inclusion and exclusion criteria.

### 2.11. Record of the Extracted and Collected Data Extraction

The results and conclusions of the selected full text papers were used for assembling the data, according to the aims and themes of the present revision, as listed onwards.

The following parameters were used as a method for assembling the data and then organized following the schemes:

“Author (Year)”—revealed the first author and the year of publication;

“Type of study”—indicated the method of the research;

“Sample size”—described the number of patients, animals or models examined;

“Bone substitute/membrane”—described types of bone grafts and membranes used for regeneration;

“Follow-up”—yes/no described the duration of the observed outcomes;

“Bone graft histology”—yes/no described the presence of the graft at 6–9 months follow-up control.

### 2.12. Risk of Bias Assessment

The grade of bias risk was independently considered, and in duplicate by the two independent reviewers at the moment of data extraction process. According to Moher and Higgins, this revision followed the Cochrane Collaboration’s two-part tool for assessing risk of bias and PRISMA statement [14,15].

Potential causes of bias were investigated:

- Selection bias;
- Performance bias and detection bias;
- Attrition bias;
- Reporting bias;
- Examiner blinding, examiner calibration, standardized follow-up description, standardized residual graft measurement, standardized radiographic assessment.

In this way, the possible random sequence generation, the possible allocation concealment, the possibility of blinding of participants and personnel, the possible presence of having incomplete outcome data and other biases were all considered and evaluated.

This method applied by the two reviewers was valuable for giving to each study a level of bias. Then, the selected papers were classified with low, moderate, high and unclear risk.

## 3. Results

### 3.1. Manuscript Collection

**Figure 1:**
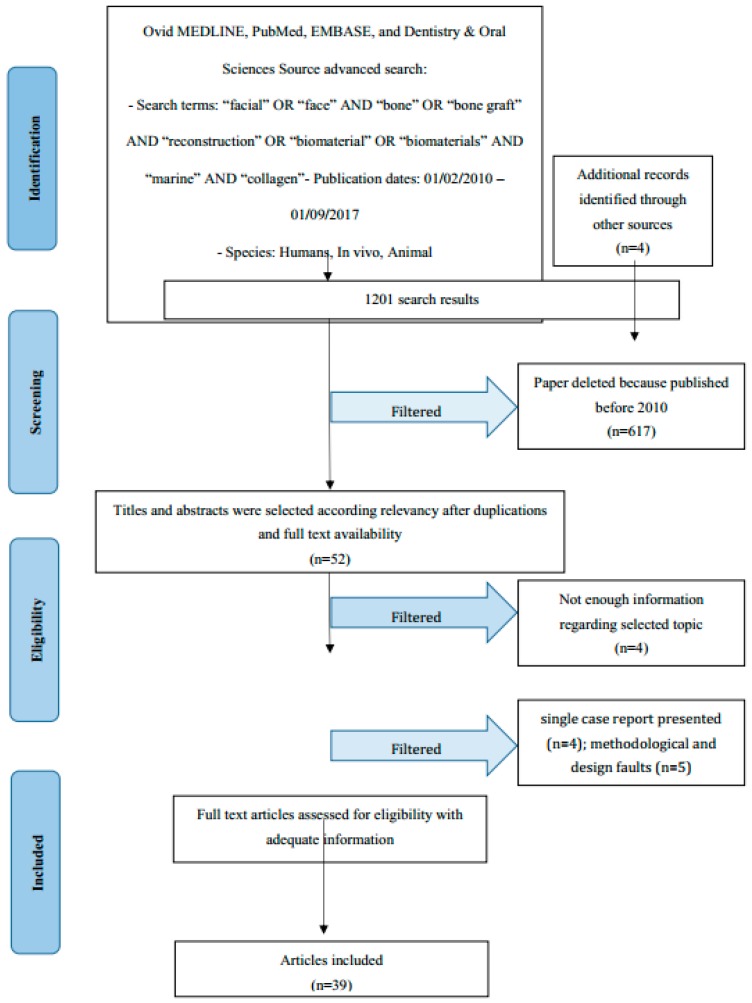
PRISMA flow diagram.

Manuscript revision and recording data process followed the PRISMA flow diagram (Figure 1). The initial electronic and hand search retrieved 1197 citations and four more papers from Dentistry, PubMed MEDLINE and Oral Sciences Source with a total of 1201 selected papers. Furthermore, 617 papers were excluded because they were published prior to 1 February 2010. Then, titles and abstracts were evaluated, and 52 articles were selected as having significant data regarding “facial bone reconstruction biomaterials” topics. In addition, 48 articles were determined as full-text papers, 39 of which were incorporated in this work. Some research was excluded due to being classified as single case reports presented (*n* = 4), weak methods or being way off-topic (*n* = 5). 

### 3.2. Study Characteristics 

After the study selection, a new division related to the kind of bone graft has been performed:

- Autlogous Bone: Thirteen studies [16,17,18,19,20,21,22,23,24,25,26,27,28];
- Homologous Bone: Five studies [29,30,31,32,33];
- Xenograft or Allograft material and bone derived from animal or synthetic origin: Ten studies [34,35,36,37,38,39,40,41,42,43];
- Marine collagen material: Eleven studies [44,45,46,47,48,49,50,51,52,53,54,55].

### 3.3. Risk of Bias within Studies

Evaluation on the total risk of bias for each selected paper, and the majority of the manuscripts were allocated as unclear risk [20,21,22,23,27,28,29,34,35,36]. Three research papers were considered as having a low risk of bias [32,37,39], where another one was classified as moderate risk [30].

### 3.4. Risk of Bias across Studies

Numerous limitations have arisen from the present revision. Current analysis of the data extracted from studies written in English only could introduce a publication bias. The main limitation of the revision is related to the different kinds of biomaterials used for the same final objective, having a bone graft material able to be predictable and safe in the reconstructive and regenerative bone tissue procedures. Regarding the bias, some selected papers have a relatively short follow-up period and, when in clinical study, included relatively small numbers of treated patients. Moreover, the presented data underlined high heterogeneity and several differences in each study method, selections of the cases, and final treatment results.

It is also important to note that choice of biomaterial for doing reconstruction surgery of the facial bones is a convoluted technique and its success is related to numerous parameters, comprehending patients’ general health conditions, oral hygiene habits, bone defect size, surgical procedures, operator skill, and various other factors that are not possible to fit within the frames of systematic literature. Table 1 resumes the studies selected and their results.

### 3.5. Autogenous Bone

Autogenous bone graft is still considered the “gold” standard graft by the international community. The selected papers underlined the autogenous bone graft osteoinductivity properity and its good integration in the treated bone defect; however, its limited availability due to the donnor site has been underlined.

Janner et al. demonstrated how the addition of autogenous bone chips and the presence of the collagen membrane increased bone formation. Wound protection from mechanical noxa during early healing may be critical for bone formation within the grafted area, but the presence of the chips guaranteed the cells osteoinduction [16]. The predictable use of autogenous bone graft has been investigated by Emodi et al. underlining the possibility of costhcondral graft application for rebuilding a mandibular condyle by using a three-dimensional printing template [17]. Moreover, the capability of autogenous graft has been proved in a split mouth study performed by Du Toit et al. Authors compared split-mouth human bone biopsy specimens derived from Platelet Rich Fibrin (PRF) and autogenous bone with bone that had healed without intervention, concluding how the quality of newly formed bone is the same in the two groups [18]. Autogenous bone graft for repairing cleft palate has been investigated by Nadon et al. The six-month outcomes of all examined patients were excellent in terms of both bone graft stability and closure of the oronasal fistulae [19]. In a retrospective study, Baden et al. evaluated the stability of bone grafted from maxillary sinus for repairing orbital trauma. The choice of autogenous bone is demonstrated to be a predictable choice for reducing the floor of orbit manually to the proper position, which helps to decrease the orbital floor defect [20]. Extra oral autogenous bone graft, and specifically the iliac bone graft for repairing the orbital floor fractures, has been retrospectively evaluated by O’Connell et al. concluding how isolated orbital blow-out fractures may be safely and predictably reconstructed using autogenous iliac crest bone [21]. Volumetric changes after autogenous ramus block bone grafting (RBG) or guided bone regeneration (GBR) in horizontally deficient maxilla before implant placement have been retrospectively evaluated by Gultekin et al. Authors stated how the two techniques are both predictable and demonstrated that the autogenous bone works have long-term dimensional stability for the next dental implant placement [22]. Zahng et al. retrospectively evaluated the outcomes of using autogenous coronoid process like bone grafts (*n* = 32) and compared with costochondral grafts (*n* = 28) in the condylar reconstruction in case of temporomandibular joint (TMJ) ankylosis. The clinical outcomes in both groups were satisfactory and comparable concluding how the autogenous coronoid process grafting may therefore be a good alternative for condylar reconstruction in patients with ankylosis of the TMJ [23]. Nkenke made a revision investigating numerous published papers about the morbidity related to the autogenous bone graft harvesting and the follow-up graft resorption and dental implant survival in the grafted sites. The author made conclusions about how the all-autologous grafts are predictable because there is no significative difference on bone resorption related to the grafting sites [24]. Cicciù et al. published a significative paper about the combination of autologous bone and growth factors applied to the mandibular continuity defects reconstruction. Authors concluded that the use of rhBMP-2 without concomitant autogenous bone grafting materials in large critical-sized mandibular defects secondary to a large mandibular tumor produced excellent regeneration of the treated area [25]. Nary Filho et al. have analyzed the possibility of autogenous bone graft infection and exposure after the regenerative surgery. The autogenous bone has less chance of causing infection due to its high possibility of integration with the host. However, all of the regenerative procedures should guarantee the covering of the grafted bone once fixed [26]. Koerdt et al. evaluated the revascularization processes in autogenous bone grafts from the iliac crest to the alveolar ridge. Even if the resorption prediction is not predictable, the immunohistochemical investigation performed showed blood vessels between the graft and the alveolar ridge [27]. Pereira Rdos et al. analyzed the management of orbital fracture with autologous bone graft in their study. A computed tomography scan shows excellent bone healing at the anterior and posterior parts of the medial orbital wall reconstruction [28].

### 3.6. Allogeneic Bone

Allogeneic bone graft used for facial reconstruction has been considered as a good alternative option in the place of autogenous bone. However, a short number of investigations recorded long-term clinical success and totally safe procedures for the treated patients.

Krasny et al. recently demonstrated how the fresh, frozen, radiation sterilized, allogeneic bone blocks constitute good and durable bone-replacement material allowing effective and long-lasting reconstruction of the atrophied alveolar ridge to support durable, implant-based, prosthetic restoration [29]. Schlee et al. underlined the concept about bone grafting with allogeneic allografts yielding equivalent results to autologous grafting, and patients appreciate the omission of bone harvesting from donor sites [30]. Monje et al. investigated the feasibility by means of survival rate, histologic analysis, and causes of failure of allogeneic block grafts for augmenting the atrophic maxilla. In their conclusions, the authors stated how the atrophied maxillary reconstruction with allogeneic bone block grafts represents a reliable option as shown by low block graft failure rate, minimal resorption, and high implant survival rate [31]. Fretwurst et al. performed a histological and biochemical evaluation of four allogeneic bone blocks concluding how this kind of graft can absolutely stimulate the newly bone formation and can be considered a valid alternative to the autologous bone graft [32]. Sbordone et al. in their study analyzed the potential bone volume changes after sinus augmentation using blocks of autogenous iliac bone or freeze-dried allogeneic bone (FDBA) from the hip. Authors concluded how application of FDBA for the short-term sinus grafting procedure showed an outcome close to that reported for autogenous bone [33].

### 3.7. Xenograft and Synthetic Bone

The bone grafts derived from animal or by synthetic production can be classified as biomaterials with osteoconductivity properties and currently have been used for being scaffolds for growth factor application in the bone defects. Scheyer et al. just published a randomized controlled multicenter clinical trial in which different biomaterials for the preservation of the bone volume after tooth extraction were tested. Authors stated how, at six months follow up, the xenograft collagen and autologous bone graft can give no significant difference on the socket modifications after extraction [34]. Le et al. evaluated 14 patients affected by soft tissue recessions around implant-supported restorations in the maxillary central or lateral incisor area. In their records, authors concluded that the use of the allograft and xenogeneic collagen significantly favored the alveolar volume conditioning hard and soft tissue dimensions in the aesthetic zone of the anterior maxilla [35]. Fienitz et al. histologically and radiologically compared a sintered and a no sintered bovine bone graft used in the sinus lift surgeries. The authors affirmed that both xenogeneic materials showed comparable results regarding the possibility of having new bone formation [36].

You et al. evaluated the effects of the bilayer bone augmentation technique for the treatment of dehiscence-type defects around implants and evaluated the role as a membrane of the xenogeneic bone using a histological method for evaluating the new bone formation. The results of this study showed the osteogenic effect of autogenous bone and the effect of mechanical support for prolonged space maintenance of xenogeneic collagen membrane applied for the treatment of dehiscence-type bone defects around implants [37]. 

De Oliveira et al. studied the regenerative results of the addition of bone marrow aspirate concentrate, using a single or double centrifugation protocol, to a xenogeneic bone graft in sinus floor elevation. This pilot study indicates that the clinical use of bone marrow aspirate concentrate, obtained by either a single or double centrifugation process, combined with a xenograft result in more adequate bone repair when used in the bone regenerative surgery [38]. Ghanaati et al. studied the structure of two allogeneic bone blocks and three xenogeneic bone grafts, which are used in dental and orthopedic surgery, and histologically analysis have been performed. The final findings affirmed that, even manufactory declared blocks were free of organic/cellular remnants, authors’ histological analysis revealed that bone blocks did contain such remnants. Moreover, such specimens might be able to induce an immune response within the recipient [39]. Peng et al. analyzed the influence of platelet rich plasma (PRP) associated with xenograft for managing peri-implant bone defects. The results indicate how the PRP associated with the bovine-derived xenograft in the small bone defect can favor the bone healing [40]. Klein et al. published a systematic histomorphometric analysis of two human bone biopsy specimens analyzed at five-years follow up after a bone regenerative procedure using a xenogeneic bovine bone substitute material. Authors demonstrated a completed bony integration without extensive resorption of the biomaterial particles [41]. Figuerido et al. evaluated the chemical and structural features of a xenogeneic and an alloplastic material highlighting the in vivo inflammatory response. 

The in vivo results analyzing the data extracted from the inflammatory infiltrates revealed that the grade of inflammation is not severe, particularly in terms of collagen production and formation of fibrous capsule [42]. 

Kim et al. investigated the efficacy of the alveolar ridge preservation technique using collagen sponge and xenograft after extraction. The results indicated that, in the ridge preservation using collagen sponge and xenograft, xenograft prevents the horizontal resorption of the alveolar ridge, and the upper collagen sponge blocks the infiltration of soft tissues to the lower area, and thus it has the advantage of the enhancement of bone fill [43].

### 3.8. Marine Collagen and Derived Bone

Marine collagen and marine derived bone substitutes as an alternative to autologous bone are quickly advancing, especially since the service of tissue engineering is researching biomaterial with low cost and high availability. Marine collagen should be a true alternative source of collagen. Marine species present a distinct advantage as a lower known risk of transmission to humans of infection-causing agents and are thought to be far less associated with cultural and religious concerns regarding the human use of marine derived products. Moreover, a clarification of the marine collagen origin is important in order to underline the microscopical features of the final material used for bone defect repairing. Not only marine invertebrate collagen sources, but also marine vertebrate ones reflect several similarity with human collagens [44]. Fish would be rich sources of collagen in terms of its production and application in various biological process. Marine organisms like coral or sponge are rich in mineralized porous structures and their microstructures seem to replace the human bone features. Evaluating the source of collagen extraction, jellyfish and invertebrate collagen are obtained from mesoglea, following a methodology based on solubilization in acetic acid solution, typically during three days. Nowadays, the collagen is considered the major constituent of the extracellular matrices of all animal and metazoans. For this reason, collagen derived from marine sponges can be evaluated as available substitutes for uses like scaffolds in the bone regenerative procedures [44,45,46,47,48,49,50,51,52].

Recently, Lin et al. developed a novel scaffold, derived from fish scales, as an alternative functional material with sufficient mechanical strength for medical regenerative applications. Fish scales, which are usually considered marine wastes, were acellularized, decalcified and fabricated into collagen scaffolds. The scanning electron microscope (SEM) was used for imaging the microstructure of the scaffold. The highly centrally-oriented micropatterned structure of the scaffold was beneficial for efficient nutrient and oxygen supply to the cells cultured in the three-dimensional matrices, and therefore it is useful for high-density cell seeding and spreading [45]. Hayashi et al. evaluated the biomedical application of chitosan and collagen from marine products and advantages and disadvantages of regeneration medicine, demonstrating that the properties of biocompatibility and biodegradation of fish atelocollagen are suitable for the scaffolds in regenerative medicine [46]. Senny et al. investigated the HE800 exopolysaccharide (HE800 EPS) secreted by a deep sea hydrothermal bacterium displays an interesting glycosaminoglycan-like feature resembling hyaluronan, and the results of the study proved how the HE800 EPS family can be considered as an innovative biotechnological source of glycosaminoglycan-like compounds useful to design biomaterials and drugs for tissue engineering applications [47]. Fernandes-Silva et al. analyzed the possibility to fabricate marine collagen porous structures cross linked with genipin under high pressure carbon dioxide. By the in vitro data results of their investigation, authors concluded that cell culture tests performed with a chondrocyte-like cell line showed good cell adherence and proliferation, which is a strong indication of the potential of these scaffolds to be used in tissue cartilage tissue engineering [48]. Yamamoto et al. published an in vitro and in vivo biological study of medical materials to investigate the safety and the predictable results on applying fish collagen for regenerative procedure. The extract of fish collagen gel was examined to clarify its sterility and demonstrated that atelocollagen prepared from tilapia is a promising biomaterial for use as a scaffold in regenerative medicine [49]. Hayashi et al. demonstrated the contributions for a proteomic view of chitosan nanoparticle to hepatic cells, the promotion of D-glucosamine to transfection efficiency, and chitin application as skin substitutes. Moreover, the latter showed the contributions for hydroxyapatite-gelatin nanocomposite, genipin modification of dentin collagen, dentin phosphophoryn/collagen composite for dental biomaterial, and biological safety of fish collagen [50]. Silva et al. evaluated all of the available forms of marine collagen and their potential application in regenerative medicine. Authors concluded that marine collagen could be considered a valuable source of collagen [51]. Jiridi et al. studied the structural and rheological properties of collagen-based gel obtained from cuttlefish skin, and to investigate its ability to enhance wound healing, demonstrating that cuttlefish collagen based gel might be useful as a wound healing agent [52]. Derkus et al. described the sonochemical isolation of nano-sized spherical hydroxyapatite (nHA) from egg shell and application towards thrombin aptasensing. The data of the presented paper reflected how, for clinical application of the developed aptasensor, thrombin levels in blood and cerebrospinal fluid (CSF) samples obtained from patients with Multiple Sclerosis, Myastenia Gravis, Epilepsy, Parkinson, polyneuropathy and healthy donors were analyzed using both the aptasensor and commercial ELISA kit. The results showed that the proposed system is a promising candidate for clinical analysis of thrombin [53]. 

Raffery et al. tried to determine if the incorporation of chitosan into collagen scaffolds could improve the mechanical and biological properties of the scaffold. In addition, the study assessed if collagen, derived from salmon skin (marine), can provide an alternative to collagen derived from bovine tendon (mammal) for tissue engineering applications. The data results underlined how the collagen–chitosan composites showed similar results to the bovine one. Moreover, a clear support to stem cell differentiation towards both bone and cartilage tissue was demonstrated. The collagen obtained from the bovine bone resulted in a versatile scaffold incorporating the marine biomaterial chitosan and showing great potential as appropriate platforms for promoting orthopaedic tissue repair while the use of salmon skin-derived collagen may be more suitable in the repair of soft tissues such as skin [54]. 

Coelho et al. performed an investigation in which collagen has been isolated from the skins of the squids using acid-based and pepsin-based protocols, with the higher yield being obtained from I. The produced collagen was selected for evaluating its biomedical potential, exploring its incorporation on poly-ε-caprolactone (PCL) 3D printed scaffolds for the development of hybrid scaffolds for tissue engineering, exhibiting hierarchical features [55].

## 4. Discussion

Nowadays, the autologous bone is considered to be the “gold standard” by the scientific community, as its use is a predictable practice. As widely described above, it increases the formation of new bone near defects. The main advantage, moreover, is to have an osteoinductive power, and to offer increased resistance to infections due to its biological activity and the revascularization of the graft, so this is a biological active tissue. In any case, in the case of autologous bone regenerations, the patient’s morbidity should be considered, due to the donor site, such as the inability to access large amounts of bone tissue [1,8,12,13,14,15,16,17,18,19,20,21,22,23,24,25,26,27,28]. However, allogeneic bone grafting is a viable alternative that has the advantage of not requiring a donor site, in the event that no alloplastic and autologous mixture grafts are used. The alloplastic material can be subjected to different chemical-physical treatment, satisfying also the requirements of larger graft surgeries, having bone blocks available [29,30,31,32,33,34]. This biomaterial also has the advantage of being able to stimulate the formation of new bone. Moreover, tissue grafts from other species or synthetic biomaterial with osteoconductivity can be available. These are mainly used as a scaffold for the regeneration of bone defects. Even if this type of material is “free cell tissue”, it could induce an immune response within the recipient [2,7,35,36,37,38,39,40,41,42,43]. Finally, marine collagen and derived bone can be considered a viable alternative, thanks mainly to its microscopic structure that would favor the metabolism of new bone tissue and hence its formation. It is similar to those found in the human spongiform bone and thus has osteoconductive abilities [45,46,47,48,49,50,51,52,53,54,55]. The porosity characteristics reflect the optimal ones, and the compression resistance is high but has little resistance to tension. As already mentioned by some studies, the ability of some cell lines to adhere to this material and proliferate on it has emerged. It is an easy-to-find material and is considered commercially as a waste material. Its production involves decellularization and then decalcification having biocompatibility properties, reducing the risk of transmitting infectious agents to humans. Marine collagen can be derived from different animal genera, from marine sponges to salmon skin or fish scales. Surely, a production of these materials using existing 3D printing technologies could revolutionize the field of biomaterials and used materials for bone facial defects.

Furthermore, Rahman published a paper just recently that demonstrated the potential for marine calcifiers to generate new drugs. Among the different sources of polysaccharides, algal polysaccharides such as chitin and collagen could play an important role in future development of tissue engineering, bone regeneration, and much more. In light of these emerging findings, in the near future, established techniques might also be potentially useful for isolating skeletal proteins from similar marine calcifiers for drug discovery [56]. Moreover, the isolation, biochemical and biophysical features of the collagen from the marine sponges Axinella cannabina and suberites carnosus were analyzed by Tzivileka et al. Authors demonstrated how marine collagen can be considered a valuable and safe alternative to the common collagen used in the current biomedical application [57]. Moreover, Hermann Ehrlich performed several experiments demonstrating how chitin and collagen are valid alternative template scaffolds in the field of mineralization [58,59,60]. Therefore, in a monograph published in 2015, the author classified significant information regarding the modern knowledge on biomineralization, biomimetics and materials science with a deep investigation about marine vertebrates. For the first time in scientific literature, the author gives the most coherent analysis of the nature, origin and evolution of biomaterials and biopolymers isolated from marine sources. Moreover, the variety of marine vertebrate organisms (fish, reptilian, birds and mammals) and within their unique hierarchically organized structural formations has been highlighted [61]. This can be a reference for performing future studies and research about the possibility of using chitin and collagen marine in the field of the biomedicine.

### Limitations

Even if a comprehensive and complete investigation of the effects of surgical therapies had been performed, there were some limitations to this systematic review. Our findings could not provide the ideal material for the bone reconstruction technique of the face. The choice of the materials depend on the size of the defect, the skill of the surgeons and the donor host in the case of autogenous bone. Moreover, there could be potential language bias in this systematic review, as we only considered literature written in English.

## 5. Conclusions

The first aim of this revision is to highlight the evolving role of biomaterial used like bone graft in the place of autologous bone during craniofacial bone reconstruction and regenerative procedures. Discussion highlighted how the ideal biomaterial scaffold is still far from being realized because of it it still being hard to reproduce the design, the micro shapes and the chemical features of the autologous bone. While human clinical applications are limited to date, great promise seems to start from scaffolds originating from the lab or obtained from the sea. Specifically, marine collagen replacing the human bone features can be optimally used for being a safe scaffold on large bone reconstruction defects of the facial area.

## Figures and Tables

**Table 1:** Studies findings and results kind of bone graft and possible clinical application.

Author and Year	In Vivo vs. In Vitro	Kind of Graft	Follow up after 12 Months
Janner et al., 2016	In Vivo	Autologous	Yes
Emodi et al., 2017	In Vivo	Autologous	Yes
Du Toit et al., 2016	In Vivo	Autologous	Yes
Nadon et al., 2015	In Vivo	Autologous	Yes
Bande et al., 2014	In Vivo	Autologous	Yes
O’Connell et al., 2014	In Vivo	Autologous	No
Gultekin et al., 2016	In Vivo	Autologous	Yes
Zhang et al., 2014	In Vivo	Autologous	Yes
Nkenke et al., 2014	In Vivo	Autologous	Yes
Cicciù et al., 2014	In Vivo	Autologous	No
Nary Filho et al., 2014	In Vivo	Autologous	Yes
Koerdt et al., 2013	In Vivo	Autologous	Yes
Pereira R et al., 2013	In Vivo	Autologous	Yes
Krasny et al., 2015	In Vivo	Homologous	Yes
Schlee et al., 2014	In Vivo	Homologous	No
Monje et al., 2014	In Vivo	Homologous	No
Fretwurst et al., 2014	In Vivo	Homologous	No
Sbordone et al., 2014	In Vivo/In Vitro	Homologous	Yes
Scheyer et al., 2016	In Vivo/In Vitro	Xenograft/Synthetic	No
Le et al., 2016	In Vivo/In Vitro	Xenograft/Synthetic	Yes
Fienitz et al., 2017	In Vivo/In Vitro	Xenograft/Synthetic	N/A
You et al., 2016	In Vivo/In Vitro	Xenograft/Synthetic	No
De oliveira et al., 2016	In Vivo/In Vitro	Xenograft/Synthetic	Yes
Ghanaati et al., 2014	In Vivo/In Vitro	Xenograft/Synthetic	Yes
Peng et al., 2016	In Vivo/In Vitro	Xenograft/Synthetic	Yes
Klein et al., 2014	In Vivo/In Vitro	Xenograft/Synthetic	No
Figueiredo et al., 2013	In Vivo/In Vitro	Xenograft/Synthetic	No
Kim et al., 2011	In Vivo/In Vitro	Xenograft/Synthetic	No
Lin et al., 2010	In Vivo/In Vitro	Marine Collagen	N/A
Hayashi et al., 2012	In Vivo/In Vitro	Marine Collagen	N/A
Senni et al., 2013	In Vitro	Marine Collagen	N/A
Fernandes et al., 2013	In Vitro	Marine Collagen	N/A
Yamamoto et al., 2014	In Vitro	Marine Collagen	N/A
Hayashi et al., 2014	In Vivo/In Vitro	Marine Collagen	N/A
Silva et al., 2014	In Vivo/In Vitro	Marine Collagen	N/A
Jridi et al., 2015	In Vivo/In Vitro	Marine Collagen	N/A
Derkus et al., 2016	In Vivo/In Vitro	Marine Collagen	N/A
Raftery et al. 2016	In Vivo/In Vitro	Marine Collagen	N/A
Coelho et al. 2017	In Vivo/In Vitro	Marine Collagen	N/A
